# A national survey of clinical pharmacy services in county hospitals in China

**DOI:** 10.1371/journal.pone.0188354

**Published:** 2017-11-30

**Authors:** Dongning Yao, Xiaoyu Xi, Yuankai Huang, Hao Hu, Yuanjia Hu, Yitao Wang, Wenbing Yao

**Affiliations:** 1 State Key Laboratory of Quality Research in Chinese Medicine, Institute of Chinese Medical Sciences, University of Macau, Macao, China; 2 School of International Pharmaceutical Business, China Pharmaceutical University, Nanjing City, Jiangsu Province, China; University of Toronto, CANADA

## Abstract

**Background:**

Clinical pharmacy is not only a medical science but also an elaborate public health care system firmly related to its subsystems of education, training, qualification authentication, scientific research, management, and human resources. China is a developing country with a tremendous need for improvements in the public health system, including the clinical pharmacy service system.

**Objectives:**

The aim of this research was to evaluate the infrastructure and personnel qualities of clinical pharmacy services in China.

**Setting:**

Public county hospitals in China.

**Materials and method:**

A national survey of clinical pharmacists in county hospitals was conducted. It was sampled through a stratified sampling strategy. Responses were analyzed using descriptive and inferential statistics. The main outcome measures include the coverage of clinical pharmacy services, the overall staffing of clinical pharmacists, the software and hardware of clinical pharmacy services, the charge mode of clinical pharmacy services, and the educational background, professional training acquisition, practical experience, and entry path of clinical pharmacists.

**Results:**

The overall coverage of clinical pharmacy services on both the department scale (median = 18.25%) and the patient scale (median = 15.38%) does not meet the 100% coverage that is required by the government. In 57.73% of the sample hospitals, the staffing does not meet the requirement, and the size of the clinical pharmacist group is smaller in larger hospitals. In addition, 23.4% of the sample hospitals do not have management rules for the clinical pharmacists, and 43.1% do not have rational drug use software, both of which are required by the government. In terms of fees, 89.9% of the sample hospitals do not charge for the services. With regard to education, 8.5% of respondents are with unqualified degree, and among respondents with qualified degree, 37.31% are unqualified in the major; 43% of respondents lack the clinical pharmacist training required by the government. Most respondents (93.5%) have a primary or medium professional title. The median age and work seniority of respondents are 31 and four years, respectively. Only 18.5% of respondents chose this occupation by personal consideration or willingness.

**Conclusions:**

The main findings in this research include the overall low coverage of clinical pharmacy services, the low rate of clinical pharmacy service software, hardware, and personnel as well as a wide variance in educational training of pharmacists at county hospitals.

## Introduction

Clinical pharmacy is a health science discipline in which pharmacists provide pharmaceutical care that optimizes medication therapy and promotes health, wellness, and disease prevention [1]. With extensive development over the past few decades, especially the global introduction of the pharmaceutical higher education and pharmaceutical service, the definition of clinical pharmacy has expanded to encompass more than a medical science, it incorporates an elaborate public health care service system firmly related to its education, training, qualification authentication, scientific research, public management and human resource subsystems [2, 3]. In other words, the evaluation of the current status of clinical pharmacy involves not only the scientific advances, but also all the aspects mentioned above [4–6]. Thus, in this essay, clinical pharmacy is discussed as an issue of public health management [7].

The clinical pharmacy service system is also involved in the development plan of nations. Initially, in 1948, the American Association of Colleges of Pharmacy first suggested introducing the clinical pharmacy education system and clinical pharmacists in the hospitals for rational drug use. The *Practice Guidelines for Pharmacotherapy Specialists*, which clarified the working content, pattern, and objectives of clinical pharmacists elaborately and systemically, was issued in 1990 and amended in 2000 [8]. It is the first management regulation that regards clinical pharmacy as a service and occupational system, and its definition of clinical pharmacy service remains a significant reference for the public health administration and clinical practice today [9].

China, as a developing country with the world’s largest population and the second largest economy, also has a progressive demand for clinical pharmacy services [10]. Clinical pharmacy was introduced in China during the 1950s and 1970s. At this time, the major duty of hospital pharmacists was medicine supply and dispensing because of the shortage of medical products in the whole public health system. Over the next few decades, the Chinese pharmaceutical industry advanced remarkably, which allowed the pharmacists to put more efforts into their patients, focusing on rational drug use. Meanwhile, the *Ordinance of Hospital Pharmacy*, the first governmental document that includes administrative rules related to clinical pharmacy, was issued by the Ministry of Health of China in 1981 [11]. In the new millennium, clinical pharmacy made a substantial development in China, with its basic education, training, and administrative systems. The *Rules of Pharmaceutical Affairs Management of Medical Institutions* (*Rules of Pharmaceutical Affairs*) issued in 2002 and amended in 2011 officially required patient-centered pharmacy services provided in hospitals, which defined the clinical pharmacists as participants of diagnosis and treatment [12, 13]. Generally, the clinical pharmacy practice system of China is developing steadily. Meanwhile, some inevitable problems are also occurring in China, including an insufficient workforce, lack of highly educated professionals, incompatibility between clinical pharmacists and physicians caused by expertise barrier, misunderstanding of the professional orientation of clinical pharmacists by decision-makers and patients, etcetera. As mentioned above, clinical pharmacy is beyond a medical science; it is an elaborate public health service system. Therefore, for its advancement, studies of social or governmental administration perspectives are required, and this kind of research, initially, requires a comprehensive understanding of the current status of clinical pharmacy practice [14–16].

The literature retrieval of clinical pharmacy or the hospital pharmacy system in China in several journal databases indicated a lack of studies in this country. There was no comprehensive and substantial conclusion on the current status. Most of the literature on China just focuses on one aspect of the whole picture [17–19] or a small geographic scale [19, 20]. County is the primary unit of the administrative region and the governmental system in China, as well as the most commonly studied level of Chinese administrative regulation. A county hospital is a public hospital that is founded, financed, and regulated by the county-level government that covers the county territory, providing basic medical services [21]. As required in related policies of the public medical system, the county hospitals provide diagnosis and medication against common diseases and frequently occurring diseases, implement resuscitation against acute and severe diseases, transfer patients with difficult miscellaneous diseases to medical institutions in the upper class, and take the duty of nursing care to patients with diagnosed chronic diseases transferred from other medical institutions [22]. On the other hand, according to data from 2014, the proportion of county hospitals to public hospitals is approximately 47.8%, and the county hospitals cover about 34.0% of the health services provided by public hospitals, which are the major medical service providers in China. Regarding the difference in size, available resources, and the functions between county hospitals and other types of public medical institutions, the county hospital has a relatively key role in the public health system of China [23]. Considering all of the reasons above, county hospitals are frequently the research object of studies on policies and regulations of China, especially concerning public health. Clinical pharmacy services are provided in most county hospitals, so, taking the role of the county hospitals and their medical coverage into consideration, the current status of clinical pharmacy services and pharmacists in county hospitals, to some extent, represents the condition of the entire health system of China. Thus, it can be a starting point for more comprehensive or deeper studies.

### Aim of the study

The purpose of this research is to assess the current status of the clinical pharmacy service system in county hospitals, using a national questionnaire survey based on stratified sampling, in order to form an accurate, comprehensive, and valuable understanding. Based on the conclusions, suggestions will be provided to the main bodies involved in the practice and related administrators.

## Materials and methods

### Design of the questionnaires

A questionnaire was designed for the clinical pharmacists of county hospitals (See S1 Survey and S2 Survey). The design of the questionnaire items was based on a literature review of related research, the current situation of clinical pharmacy practice in China, and the aim of this study. The questionnaires were structured to mainly cover the following items: (1) basic information about the medical institution; (2) the coverage of the clinical pharmacy service; (3) the status of the clinical pharmacy service software and hardware; (4) the charge mode of the clinical pharmacy service; (5) the educational background of the clinical pharmacists; (6) the professional training of the clinical pharmacists; (7) the practical experience of the clinical pharmacists; and (8) the entry path of the clinical pharmacists. Each aspect above involves two or three specified variables.

A pilot survey of 5 county hospitals in Nanjing of Jiangsu province was conducted in June 2015 as a test for the designed questionnaire, sampling, and survey. 3 clinical pharmacists of each pilot hospital were included in the sample, and the sample size is 15 in total. Additionally, a face-to-face interview of the hospital director(s) of each pilot hospital was launched by our researchers to collect valuable comments and advice on the pilot survey questionnaire. Accordingly, some detailed adjustments of the questionnaire were made.

### Sampling of the medical institutions

Geographically, China is the third largest country in the world, with a population of over 1.35 billion, and its public health system is correspondingly large and intricate. The aim of this survey is to describe the status of the clinical pharmacy practice of county hospitals in the whole country. Thus, a set of complex sampling procedures based on a classification of per capita disposable income (PCDI) were designed to ensure the representativeness of the sample:

1. Stratified sampling: all the counties in a single provincial administrative unit were divided into three groups of equal number of members by PCDI to ensure that our sample covers all economic levels in China.
2. Convenience sampling: within each group, the number of sampled county hospitals was equal to a third of the total number of the counties in this group, and the specific sampled hospitals were selected according to the accessibility of their information and the convenience of our actual survey.
3. Quantity of questionnaire collection: within each of the sample hospitals, at least one questionnaire was collected, and the investigators were required to collect as many questionnaires as accessible.

By the sampling methods above, 317 medical institutions from all 31 provincial administrative units in mainland China were selected to be the sample institutions of this survey. Hong Kong, Macau, and Taiwan were excluded because the public health system of these three provincial administrative regions is distinctively different from the system applied in mainland China.

We contacted the director of each county hospital in the sample with telephone or face-to-face conversation to make a brief introduction of the aim, the content and the survey plan of our research. A group of investigators would be sent to a hospital to when verbal consent of further discussion is acquired from the hospital director. It is fortunate that all directors of hospitals in the sample were supportive to our survey.

### Data collection and analysis

All data in this research were collected through field questionnaire surveys (See S1 Data). Undergraduate students with a higher education background in pharmacy or pharmaceutical science were recruited for data collection. All investigators were well trained by our research team and appropriate professionals.

The survey was conducted during July and August 2015. Each investigator was appointed to cover two or three sampled hospitals. An informed consent form was provided to the director of each sample hospital and clinical pharmacists involved in the survey, and the survey was conducted after acquiring written informed consent of each person above. To ensure the validity and quality of the data, all questionnaires were administered through an interview survey. Collected data was examined by our researchers, and for data with flaws or missing data, return interviews were conducted.

According to the *Rules of Pharmaceutical Affairs*, a county hospital is required to establish a pharmaceutical service team comprising three clinical pharmacists as the minimum. Thus, the planned questionnaire quantity of our survey is five for each sample hospital. In order to acquire valid basic information of the medical institution, the investigators are required to ensure that at least one of the respondents within a single sample hospital is a member of the leadership of the pharmacy department or the hospital. If the information varied across the collected questionnaires of one hospital, a return interview would be conducted for unified information. The number of valid questionnaires collected was 357, and the usage rate of the questionnaire was 22.52%. The usage rate is relatively low because of the low accessibility of the clinical pharmacists caused by their temporary absence from workplace or the shortage of clinical pharmacist staff of the hospitals. The intention of collecting as many valid questionnaires as possible, for which we provided expanded number of blank questionnaire, is another cause of low usage rate. Nonetheless, the sample still covered each hospital elected through the sample procedures above, which, to some extent, geographically ensured representativeness of the sample.

Due to the complexity of the questionnaire variables and large sample size, a double entry and validation method was applied based on EPIDATA to ensure the correctness and effectiveness of data entry, specifically that it realized the constraint and validation of the type, missing value, range and correction of repeated entry of raw data. All data were analyzed using descriptive and inferential statistical methods. Indicators of coverage of clinical pharmacy service and age and work seniority of clinical pharmacists are analyzed with the minimum, lower quartile, median, upper quartile and maximum values among all sample hospitals; indicators of the overall staffing of clinical pharmacists are analyzed with the mean values of hospitals of different sizes; indicators of the software and hardware of clinical pharmacy services are analyzed with percentages of each item in all sample hospitals (n = 317); current status of clinical pharmacists (except for age and work seniority) are analyzed with percentages of each item in all sample hospitals (n = 357). In issues that number of sample hospitals is used as total case number, we checked and reconfirmed collected data to ensure that multiple data provided by clinical pharmacists of same hospital are unified.

The data was processed and formed by SPSS 24 (IBM Corporation, Armonk, NY, USA), which is compatible with Microsoft Office software products in statistical figure and table output.

### Ethics approval

The approval to conduct the pilot survey and main survey was granted by the Ethics Committee of the China Pharmaceutical University. Project number: CPU2015006.

## Results

### Current status of the clinical pharmacy service system

This section of the results contains a series of data on the current status of the clinical pharmacy service system of the sample hospitals, which is based on the level of service coverage, the software and hardware, and the staffing of clinical pharmacists.

### Coverage of the clinical pharmacy service

A county hospital in China is required to establish a medical team that consists of physicians, pharmacists, and nurses, which can be interpreted as a demand for 100% coverage of clinical pharmacy services on both the department scale and the patient scale. The minimum value, lower quartile, median, upper quartile, and maximum value were applied to describe coverage of the service in the two scales, as shown in Table 1.

**Table 1:** Coverage of clinical pharmacy services.

	Department coverage^a^	Patient coverage^b^
Min	5.62%	2.57%
Lower quartile	8.31%	10.45%
Median	18.25%	15.38%
Upper quartile	20.32%	21.06%
Max	34.22%	28.71%

^a^ Coverage on the department scale is measured by the proportion of clinical departments providing a clinical pharmacy service among all clinical departments.

^b^ Coverage on the patient scale is measured by the proportion of patients who received clinical pharmacy service among all outpatients and inpatients.

Coverage of the clinical pharmacy service on both the department scale and the patient scale is extremely low (as shown in Table 1). The medium values of both are lower than 20%, and the maximum values of both are lower than 35%. This is far from the requirements specified in the *Rules of Pharmaceutical Affairs*.

### The overall staffing of clinical pharmacists

This part of the survey focuses on the staffing of clinical pharmacists in the county hospitals. This refers to the number of beds and the number of pharmaceutical service staff, which are key indicators for measuring the quantity and even the quality of the services. The number of clinical pharmacists is especially important. According to the *Rules of Pharmaceutical Affairs*, a pharmaceutical service group must have at least three full-time clinical pharmacists in a county hospital. However, our survey data shows that the medium number of clinical pharmacists of the sample hospitals is two, which means more than half of the county hospitals are not up to the standard established by the government. Furthermore, the ratio between the number of sickbeds and full-time clinical pharmacists and the ratio between the number of pharmaceutical professionals and full-time clinical pharmacists can indicate the actual capability, efficiency, and quality of the clinical pharmacy services. The data in Table 2 shows the proportional relations between the number of sickbeds, the pharmaceutical professionals, and the clinical pharmacists of each sample hospital.

**Table 2:** Relative quantity of clinical pharmacists.

Number of sickbeds	0–50	51–100	101–200	201–500	>501
Percentage	4.39%	0.98%	6.83%	32.44%	55.37%
Mean value: sickbeds	25(±8.76)	100(±0.00)	151.43(±32.46)	402.84(±94.15)	788.43(±503.58)
Mean value: pharmaceutical professionals^a^	9.22(±8.97)	9.5(±.1.27)	9.61(±6.74)	29.71(±13.01)	44.9(±258.56)
Mean value: clinical pharmacists	2.89(±1.91)	4.5(±3.58)	1.71(±1.22)	3.3(±4.41)	3.6(±4.16)
Clinical pharmacists per 100 beds	11.56	4.5	1.13	0.82	0.46
Ratio of clinical pharmacists and pharmaceutical professionals^a^	0.31	0.47	0.18	0.11	0.08

^a^ Pharmaceutical professional: including pharmacy administrators, distribution pharmacists, clinical pharmacists and pharmacy technicians.

The data indicated that the ratio of the number of full-time clinical pharmacists and the number of sickbeds is lower in larger hospitals, and so is the ratio of the number of full-time clinical pharmacists and the number of pharmaceutical professionals, generally. This can be interpreted as a decrease of the staffing level of clinical pharmacists when the hospital is sorted in ascending order.

### The software and hardware of clinical pharmacy services

Table 3 shows the equipment of management rules of the clinical pharmacists, the rational drug use software, and the charge strategies of the sample hospitals.

**Table 3:** The software and hardware of clinical pharmacy services.

	Management rules of the clinical pharmacists^a^	Rational drug use software	Charged services
No	23.4%	43.1%	89.9%
Yes	76.6%	56.9%	10.1%

^a^ Management rules of the clinical pharmacists: the management rules and guidelines for the clinical pharmacists issued and applied within the hospital.

The pharmacy department of a hospital in China is required to establish and apply rules, guidelines, and records of clinical pharmacy services and clinical pharmacists in the scale of the hospital. However, 23.4% of the sample hospitals in our survey lack functioning management rules.

Rational drug use software is also known as the sound drug formulary system, which is a drug information administration system constructed based on clinical medication databases. It functions as part of the hospital information system (HIS), providing information on diagnosis and clinical drug use. The rational drug use software has become an essential tool for the modern clinical pharmacy service. It was originally introduced in the 1980s in China, and at present, there are four types of mainstream rational drug software developed for hospitals in China. The use of rational drug use software is not specifically required by the *Rules of Pharmaceutical Affairs*, but several Chinese researchers have proved that the software elevated the quality and efficiency of clinical pharmacy service in hospitals in China [24,25], thus we included this indicator in our research. Our survey data shows that only 56.9% of the sample hospitals are equipped with the software. Thus, this may represent a potential flaw of the present clinical pharmacy system of the county hospitals.

Clinical pharmacy services are chargeable in many countries, while in China, the rules and strategies for charging for clinical pharmacy services are still in the process of discussion and construction. According to our data, the services provided by 89.9% of respondents are not charged, and among the charged services, pharmaceutical care/monitoring and pharmaceutical consultation account for the largest proportions.

### Current status of clinical pharmacists

The clinical pharmacist is the actual provider of the clinical pharmacy service. Their professional competence and personal qualities are decisive factors in the service quality. This part of the results shows the key indicators for measuring the competence and personal qualities of the clinical pharmacists.

#### Educational background of clinical pharmacists

The clinical pharmacy service, as a medical service, has high requirements about the expertise of the service providers, which can be partially measured by the educational degree and major. In America, Pharm.D is required for a clinical pharmacist, but in China, the requirement is less stringent: only a bachelor degree of pharmacy or clinical pharmacy is required. With a higher or more pharmacy-related or clinical-pharmacy-related educational background, a clinical pharmacist is assumed to be more educated, skilled, and qualified in clinical treatment practice. This makes the educational background another measure of the professional knowledge and skills of the clinical pharmacists. According to our survey data, the educational background of the clinical pharmacists varies widely.

Table 4 describes the structure of the highest education level of the respondents. The highest degree of most respondents is bachelor degree (62%). A master degree accounts for the second largest proportion (21.8%), and the proportion with a doctorate degree (7.7%) is an indication of the higher educational background requirements for clinical pharmacists. Nevertheless, it is also obvious that 8.5% of respondents have an unqualified degree.

**Table 4:** Highest education level of clinical pharmacists.

College degree	8.5%
Bachelor degree	62%
Master degree	21.8%
Doctor degree	7.7%

On the other hand, as displayed in Table 5, the respondents’ majors of both first degree and the highest degree vary greatly. Nine percent of respondents have first degrees in majors related to medical science, and the same proportion for the highest degree is 5.1%. Regarding the highest degree, only 10.1% of respondents have a degree in clinical pharmacy, and 42.4% have a degree in pharmacy, which means 47.5% of the clinical pharmacist respondents do not meet the qualifications listed in the *Rules of Pharmaceutical Affairs*.

**Table 5:** Major of degree of clinical pharmacists.

Major of first degree		Major of highest degree	
Pharmacy	51.0%	Pharmacy	42.4%
Clinical pharmacy	13.3%	Clinical pharmacy	10.1%
Clinical medicine	4.5%	Clinical medicine	5.1%
Pharmaceutics	6.6%	Pharmaceutics	5.8%
Pharmacology	5.9%	Pharmacology	8.6%
Pharmaceutical chemistry	4.5%	Pharmaceutical chemistry	5.8%
Pharmaceutical engineering	4.5%	Pharmaceutical engineering	5.1%
Traditional Chinese medicine	5.2%	Traditional Chinese medicine	6.5%
Combination of Chinese traditional and Western medicine	4.5%	Dosimetry	5.1%
		Pharmaceutical marketing	5.1%

#### Professional training acquisition

An official clinical pharmacist training system that is classified into two categories (provincial and national) and four professions (generalized and specialized for each category) is applied in China as an occupational requirement. The respondents’ acquisition of professional training is displayed in Table 6.

**Table 6:** Professional training status.

National specialized	35.8%
National generalized	9.5%
Provincial specialized	11.7%
Untrained^a^	43%

^a^ untrained: without any official clinical pharmacist training of any level or type.

It is noteworthy that 43% of respondents are without any qualification. The respondents with qualifications are basically holding the national specialized qualification.

#### Practical experience of the clinical pharmacists

Professional title is a qualification system applied in China as an assessment of the professional skills and knowledge of professionals regarding their specific domain.

The majority of respondents have a primary title (43.8%) and a medium title (49.7%) (as shown in Table 7). This can be interpreted as an insufficiency of highly skilled and educated professionals among clinical pharmacists in the county hospitals.

**Table 7:** Professional title of clinical pharmacists.

Primary title (assistant pharmacist and pharmacist)	43.8%
Medium title (pharmacist-in-charge)	49.7%
Sub-senior title (associate chief pharmacist)	3.7%
Senior title (chief pharmacist)	2.8%

Age and work seniority are both important indicators for measuring work experience, which to some extent, determines the quality of the clinical pharmacy services. Table 8 shows that the majority of clinical pharmacist respondents in our survey are aged between 28 and 38.75 and they have work seniorities of between two and five years, which indicates a universal lack of work experience among clinical pharmacists.

**Table 8:** Age and work seniority.

	Age (year)	Work seniority^a^ (year)
Minimum	24	0
Lower quartile	28	2
Medium	31	4
Upper quartile	38.75	5
Maximum	50	26

^a^ Work seniority is the accumulated total number of years of working as a full-time clinical pharmacist in the medical institution(s) of a respondent.

#### The entry path for clinical pharmacists

The path for entering the clinical pharmacist career may affect the initiative and occupational development of a clinical pharmacist, thus we included it in our survey to evaluate the future pattern of clinical pharmacists. The proportions of three major entry paths that the respondents took are shown in Table 9.

**Table 9:** Entry paths of clinical pharmacists.

By assignment of the hospital^a^	47.3%
By personal consideration	18.5%
By cultivation program^b^ of college or university	20.7%
Other	13.6%

^a^ By assignment of the hospital: In China, hospitals may initiate training projects or provide opportunities to pharmacists, medical care personnel, or other types of employees to develop clinical pharmacy professionals.

^b^ By cultivation program: In China, most colleges or universities with medical or pharmaceutical majors are contracted with medical institutions, which provide internship and formal posts to the graduates as an efficient path for the training of medical or pharmaceutical science personnel.

Almost half (47.3%) of the respondents gained their position in the clinical pharmacist group by hospital assignment. Meanwhile, 20.7% of respondents gained their position from a cultivation program of the higher education system. Both of these methods are passive entry paths, which may lead to a lack of willingness for long-term career development. In contrast, the active path accounts for only 18.5% according to the data.

## Discussion

This is the first national survey of clinical pharmacy services in China. The original purpose of this survey was to report the basic status of clinical pharmacy services in county hospitals, but several intriguing facts were discovered based on the survey data. These facts were discussed in regard to present conditions and administrative measures in China, and they can be valuable references for other countries encountering similar problems.

### Low rate of clinical pharmacy service software, hardware, and personnel

Infrastructure, which consists of software, hardware [26], and personnel [27], determines the accessibility and quality of the clinical pharmacy service to some extent [28]. Our research specified the infrastructure status of the clinical pharmacy service system of Chinese county hospitals according to several indicators, which are comparable to related administrative rules or statistics. All the data suggest that there is insufficient software establishment, hardware equipment, and clinical pharmacy staffing.

In China, a county hospital is required to establish a clinical pharmacist group with more than three clinical pharmacists, and according to our survey data, most hospitals have met this standard. However, the *Rules of Pharmaceutical Affairs* also suggests that the actual size of the clinical pharmacy service group should be adjusted according to the size, function, and population coverage of the hospital. Thus, an imbalance in the staffing of clinical pharmacists among the county hospitals of different sizes was detected. The clinical pharmacist group of most sample hospitals barely reached the specifically required size of three in larger county hospitals. With a significantly lower density of clinical pharmacists (referring to the bed numbers and size of the pharmaceutical staff), this may seriously harm the accessibility and quality of the clinical pharmacy services. This condition can be discussed from multiple aspects. Within our research, it can be linked to the problem of occupational confidence and future expectation, which might be attributable to the absence of charged services.

The independent charged service item is officially proposed in the next phase of the medical system reform of China. However, at present, the lack of a standardized pricing and charging strategy for clinical pharmacy services and the consequent uncharged services may lead to problems in evaluating the effectiveness and value of clinical pharmacy services. In turn, this may lower the occupational confidence and future expectation of the clinical pharmacists, according to international experience [29]. The services provided by most respondents are not charged, and this is a potential explanation for the imbalance between the clinical pharmacist group size and the hospital size. Low occupational confidence and future expectation is also reflected in the data of the entry path of the clinical pharmacists. Although the higher education system and the hospital system are putting considerable efforts into clinical pharmacist cultivation, only a minor proportion of clinical pharmacists in the hospitals entered this career by personal consideration.

On the other hand, the hardware (rational drug use software or health information system) and software (rules, guidelines, and records of clinical pharmacy services) seem to hamper the clinical pharmacy service. Official statistical data of 2015 shows that most county hospitals give preferential expenditure to medical hardware, services, and personnel, which means that the expense priority of a county hospital would be the expenses of primary medical service, the sustenance of the institution, and the expansion budget. This may lead to overlooking or avoiding the software and hardware of the clinical pharmacy service. Another possible explanation for the inadequacy of the software and hardware could be the hospital leaders’ misunderstanding of the importance, function, or requirements of the clinical pharmacy service.

Under the combined effect of the low rate of clinical pharmacy service software, hardware, and personnel analyzed above, the clinical pharmacy service system of county hospitals in China is facing an impediment of infrastructure construction, which is the basis for providing and developing the services properly. This explains the remarkably low coverage of service on both the department and patient scales. Improvements in the value cognition, software establishment, and hardware are urgently needed.

### Training and competence of clinical pharmacists

The clinical pharmacy service shares one characteristic with the medical service: its clinical practice relies on both specialized knowledge and practical experience of the provider[30, 31]. Therefore, a standard for the expertise of the clinical pharmacist is necessary[32]. With this consideration, both the educational/training background and the work experience of pharmacists are applied in our survey.

Requirements for educational background are clearly specified in the *Rules of Pharmaceutical Affairs*, but, referring to the data, some clinical pharmacists are unqualified in both the dimension of highest education level and major of higher education. Lack of qualification in the major seems particularly obvious. With an individual case analysis, we found that over 35% of respondents had not acquired degree of pharmacy or clinical pharmacy, which is an expressly stated requirement for the entry of a clinical pharmacist in China, within their entire higher education period. Some information provided in related research revealed differently oriented problems about this condition. The insufficient base number of clinical pharmacy personnel is the first factor brought up by the respondents. There is a large deficiency of professionals provided by the preset training system. Secondly, given that the county hospital system plays an important role in the whole public health system, they can hardly provide attractive wages and working conditions matching qualified clinical pharmacy personnel, who prefer superior leveled medical institutions. In attempting to reach the quantitate requirement of clinical pharmacists by the rules, county hospitals may resort to employing unqualified personnel. Another cause of lack of qualification is that the definition of a major in pharmacy can be misinterpreted by hospital leaders. The term ‘major of pharmacy’ is possibly interpreted to be pharmacy related major. Under most circumstances, the conditions above occurred spontaneously, resulting in unqualified clinical pharmacists.

The same issue also occurs in the professional training of clinical pharmacists. Although the *Rules of Pharmaceutical Affairs* mentions the requirement of standardized training of clinical pharmacists, the definition of training is vague. Besides the national and provincial training, trainings provided by hospitals or higher education institutions are also accepted as standardized trainings by county hospitals. Generally, the misinterpretation of the rules is bringing disorder to the clinical pharmacy service system. Again, the small base number is also a cause of the shortage of well trained and experienced clinical pharmacists.

The lack of work experience worsened the situation mentioned above [33]. The majority of respondents hold a primary or medium professional title and are aged around 31, with average working seniority of around five years. A comparison between clinical pharmacists and clinical physicians can be made to illustrate the condition. The average age of clinical physicians in 2015 was around 40 and the working seniority was around 20. Additionally, the majority of clinical physicians had a medium title or higher. The mismatch in the ages, working seniority levels, and professional titles could affect the cooperation between clinical pharmacists and physicians, who are intimate collaborators in clinical medication as required in the rules.

The lack of qualification and variance of educational training of clinical pharmacists reflects a systemic insufficiency and disorder of the personnel cultivation and training of the clinical pharmacy service system.

### Study limitations

There are several limitations of the present study due to uncontrollable issues and conditions. Firstly, the representativeness of county hospitals among the entire Chinese public hospital system is only valid in key elements of clinical pharmacy services. Due to significant differences in size, capability, function, and other aspects of different types of public hospitals or private hospitals, the illustration of the whole picture requires a series of studies narrowed down to specific type of hospital. Secondly, the purpose of our research being reaching a comprehensive and full-scale description of the present clinical pharmacy service system of the county hospitals in China, some issues derived from the discussion require additional data and references for further discussion and precise conclusions. For example, the reasons that leaders overlook the importance of the clinical pharmacy service, and all assumptions provided in the discussion are merely based on the limited data of our survey, the information provided in other research, or our knowledge of the current situation, but without solid supporting data.

## Conclusions

This research is a national questionnaire survey on the present state of the clinical pharmacy services provided in county hospitals in China. It shows the general condition of the entire public health system. Our findings suggest that the low equipment rate of clinical pharmacy service software, hardware, and personnel are the main hindrances to full coverage of clinical pharmacy services, whose quality is also lowered by the wide variance in educational training of pharmacists at county hospitals. This condition shows an opportunity to include more pharmacists trained in pharmacy or clinical pharmacy than other related fields such as pharmacology, and more pharmacists with higher degrees. And, as a conclusion, the clinical pharmacy service system in county hospitals of China still requires further development and improvement.

## Supporting information

S1 Survey The survey questionnaire(English Version). (DOCX)

S2 Survey The survey questionnaire(Chinese Version). (DOCX)

S1 Data Data acquisition application. (DOCX)
