# Supplementary material for: A national survey of clinical pharmacy services in county hospitals in China
Source: PLoS One. 2017 Nov 30;12(11):e0188354. doi: 10.1371/journal.pone.0188354 (PMC5708790; doi:10.1371/journal.pone.0188354)
Supplement: S1 Survey — (DOCX) [file pone.0188354.s001.docx]

| National Survey of Clinical Pharmacy Services in County Hospitals in China | | |
| --- | --- | --- |
| Interviewee_______ Phone Number______________  Questionnaire Number ______________ Name of Medical Institution__________________ City _______________ | | |
| Number | Questions | Answers |
| 1 | How many clinical departments are there in your medical institution? | ______ |
| 2 | How many clinical departments implement clinical pharmacy services are therein your medical institution? | ______ |
| 3 | What is the percentage of patients who received clinical pharmacy service in your medical institution? | ______% |
| 4 | How many clinical pharmacists are therein your medical institution? | ______ |
| 5 | How many beds in-service useare there in your medical institution? | ______ |
| 6 | How many pharmaceutical professionals are there in your medical institution? | ______ |
| 8 | Is there any management system of clinical pharmacists in your medical institution? | □Yes□No |
| 9 | Is your medical institution equipped withrational drug use system? | □Yes□No |
| 10 | Is clinical pharmacy service in your medical institution charged? | □Yes□No |
| 11 | What is your final educationaldegree? | □College□Bachelor  □ Master □Ph.D. |
| 12 | What is major in first educationaldegree? | ______ |
| 13 | What is your major of final educational degree? | ______ |
| 14 | Which kind of clinical pharmacist professional training certificate did you acquire? | □Provincial Specialist □Provincial Generalist  □National Specialist  □National Generalist |
| 15 | What is your professional title? | □Junior Title  □Middle Title  □Vice-senior Title  □Senior Title |
| 16 | What is your age? | ______ |
| 17 | How long is your work seniority of clinical pharmacist? | ______year(s) |
| 18 | How do you become clinical pharmacist? | □Orientation Training by Hospital  □Personal Career Direction  □Orientation Training by University□Others |

References

1. American College of Clinical P. The definition of clinical pharmacy. Pharmacotherapy. 2008;28(6):816-7.
2. Smith JE, Shane R. Clinical career ladders: application to hospital pharmacy practice. Am J Hosp Pharm. 1989;46(11):2259-62.
3. Smith F. The quality of private pharmacy services in low and middle-income countries: a systematic review. Pharm World Sci. 2009;31(3):351-61.
4. Zhenhua Z, Xuan L. Past, Present and future of the clinical pharmacy in China. China Prescription Drug. 2009(04):22-5.
5. Rules of Pharmaceutical Affairs Management of Medical Institutions. China Licensed Pharmacist. 2011(03):41-4.
6. Ming H, Xuehua J, Yongpei W, Qing Y, Xixi L. Current situation of hospital pharmacy services and clinical pharmacy in China (part 1) -- Investigation on general situation of pharmaceutical care in hospital. China Pharmacy. 2009(01):72-4.
7. ming H, Xuehua J, Wuyongpei, Yanqing, xixi L. Status quo of hospital pharmacy services and clinical pharmacy in China (part 2) -- Investigation on the development of clinical pharmacy. China pharmacy. 2009(13):1030-2.
8. ming H, Lingli Z, Xuehua J, Wuyongpei, Yanqing, xixi L. Current situation of hospital pharmacy services and clinical pharmacy in China (part 3) -- Investigation on attitude intention of clinical pharmacy. China Pharmacy. 2009(16):1278-80.
9. Zhu M, Guo DH, Liu GY, Pei F, Wang B, Wang DX, et al. Exploration of clinical pharmacist management system and working model in China. Pharm World Sci. 2010;32(4):411-5.
10. American Society of Hospital P. ASHP guidelines: minimum standard for pharmacies in hospitals. American journal of health-system pharmacy : AJHP : official journal of the American Society of Health-System Pharmacists. 2013;70(18):1619-30.
11. ASHP guideline: minimum standard for pharmaceutical services in ambulatory care. American Society of Health-System Pharmacists. American journal of health-system pharmacy : AJHP : official journal of the American Society of Health-System Pharmacists. 1999;56(17):1744-53.
12. ASHP guidelines: minimum standard for pharmacies in institutions. American journal of hospital pharmacy. 1985;42(2):372-5.
13. Buxton JA, Babbitt R, Clegg CA, Durley SF, Epplen KT, Marsden LM, et al. ASHP guidelines: Minimum standard for ambulatory care pharmacy practice. American journal of health-system pharmacy : AJHP : official journal of the American Society of Health-System Pharmacists. 2015;72(14):1221-36.
14. ASHP guidelines on a standardized method for pharmaceutical care. American Society of Health-System Pharmacists. American journal of health-system pharmacy : AJHP : official journal of the American Society of Health-System Pharmacists. 1996;53(14):1713-6.
