# Supplementary material for: A national survey of clinical pharmacy services in county hospitals in China
Source: PLoS One. 2017 Nov 30;12(11):e0188354. doi: 10.1371/journal.pone.0188354 (PMC5708790; doi:10.1371/journal.pone.0188354)
Supplement: S2 Survey — (DOCX) [file pone.0188354.s002.docx]

| 全国县级医院临床药学服务开展情况调查表 | | |
| --- | --- | --- |
| 受访者姓名_______联系电话______________问卷编号______________ 医疗机构名称__________________医疗机构所在城市_______________ | | |
| 序号 | 问题 | 回答 |
| 1 | 您所在医疗机构共有多少临床科室？ | ______个 |
| 2 | 您所在医疗机构中有多少临床科室开展了临床药学服务？ | ______个 |
| 3 | 您所在医疗机构接诊的患者中有多大比例接受了临床药学服务？ | ______% |
| 4 | 您所在医疗机构一共有多少名临床药师？ | ______名 |
| 5 | 您所在医疗机构一共有多少张实际使用床位？ | ______张 |
| 6 | 您所在医疗机构一共有多少名药学专业人员？ | ______名 |
| 8 | 您所在医疗机构是否有临床药师管理制度？ | □是 □否 |
| 9 | 您所在医疗机构是否使用合理用药软件系统？ | □是 □否 |
| 10 | 您所在医疗机构临床药学服务是否收费？ | □是 □否 |
| 11 | 您的最终学历是？ | □大专 □本科  □ 硕士 □博士 |
| 12 | 您的第一学历专业是？ | ______ |
| 13 | 您的最终学历专业是 | ______ |
| 14 | 您获得了何种临床药师专业培训证书？ | □省级专科 □省级通科  □国家级专科 □国家级通科 |
| 15 | 您的职称是？ | □初级职称 □中级职称  □副高级职称 □高级职称 |
| 16 | 您的年龄是？ | ______ |
| 17 | 你从事临床药师职业的年限是？ | ______年 |
| 18 | 您是如何成为临床药师的？ | □医院直接指定培养  □出于个人求职意愿  □高校定向培养  □其他 |

参考文献：

1. American College of Clinical P. The definition of clinical pharmacy. Pharmacotherapy. 2008;28(6):816-7.
2. Smith JE, Shane R. Clinical career ladders: application to hospital pharmacy practice. Am J Hosp Pharm. 1989;46(11):2259-62.
3. Smith F. The quality of private pharmacy services in low and middle-income countries: a systematic review. Pharm World Sci. 2009;31(3):351-61.
4. Zhenhua Z, Xuan L. Past, Present and future of the clinical pharmacy in China. China Prescription Drug. 2009(04):22-5.
5. Rules of Pharmaceutical Affairs Management of Medical Institutions. China Licensed Pharmacist. 2011(03):41-4.
6. Ming H, Xuehua J, Yongpei W, Qing Y, Xixi L. Current situation of hospital pharmacy services and clinical pharmacy in China (part 1) -- Investigation on general situation of pharmaceutical care in hospital. China Pharmacy. 2009(01):72-4.
7. ming H, Xuehua J, Wuyongpei, Yanqing, xixi L. Status quo of hospital pharmacy services and clinical pharmacy in China (part 2) -- Investigation on the development of clinical pharmacy. China pharmacy. 2009(13):1030-2.
8. ming H, Lingli Z, Xuehua J, Wuyongpei, Yanqing, xixi L. Current situation of hospital pharmacy services and clinical pharmacy in China (part 3) -- Investigation on attitude intention of clinical pharmacy. China Pharmacy. 2009(16):1278-80.
9. Zhu M, Guo DH, Liu GY, Pei F, Wang B, Wang DX, et al. Exploration of clinical pharmacist management system and working model in China. Pharm World Sci. 2010;32(4):411-5.
10. American Society of Hospital P. ASHP guidelines: minimum standard for pharmacies in hospitals. American journal of health-system pharmacy : AJHP : official journal of the American Society of Health-System Pharmacists. 2013;70(18):1619-30.
11. ASHP guideline: minimum standard for pharmaceutical services in ambulatory care. American Society of Health-System Pharmacists. American journal of health-system pharmacy : AJHP : official journal of the American Society of Health-System Pharmacists. 1999;56(17):1744-53.
12. ASHP guidelines: minimum standard for pharmacies in institutions. American journal of hospital pharmacy. 1985;42(2):372-5.
13. Buxton JA, Babbitt R, Clegg CA, Durley SF, Epplen KT, Marsden LM, et al. ASHP guidelines: Minimum standard for ambulatory care pharmacy practice. American journal of health-system pharmacy : AJHP : official journal of the American Society of Health-System Pharmacists. 2015;72(14):1221-36.
14. ASHP guidelines on a standardized method for pharmaceutical care. American Society of Health-System Pharmacists. American journal of health-system pharmacy : AJHP : official journal of the American Society of Health-System Pharmacists. 1996;53(14):1713-6.
