# Supplementary material for: A national survey of clinical pharmacy services in county hospitals in China
Source: PLoS One. 2017 Nov 30;12(11):e0188354. doi: 10.1371/journal.pone.0188354 (PMC5708790; doi:10.1371/journal.pone.0188354)
Supplement: S1 Data — (DOCX) [file pone.0188354.s003.docx]

“中国县级医院临床药学服务现状调研（National Survey of Clinical Pharmacy Services in County Hospitals in China）”是由中国药科大学国家执业药师发展研究中心开展的一项大型社会调研。

本调研针对于我国县级医院临床药学服务开展情况，收集、分析最新的高质量微观数据，并讨论我国县级医院临床药学服务软硬件及人员配备，服务人员素质和状态，为制定和完善我国相关政策提供更加科学的基础。本调研于2015年7月至8月间实施，基于大样本问卷调研，覆盖我国大陆地区全部31个省\自治区\直辖市，共317家县级医院。本调研结合了国内外临床药学服务相关标准和政策，以及临床药学服务调查的国际经验，在多阶段复杂抽样的基础上对数据进行了统计分析。

本调研是我国第一个覆盖整个大陆地区的关于县级医院临床药学服务的大样本调研，也是一系列相关调研的开山之作。

如需具体调查问卷和数据，请填写附表，并发至邮箱，联系我们。

| 中国县级医院临床药学服务现状调研数据获取申请表 | | | |
| --- | --- | --- | --- |
| 申请人 |  | 申请机构 |  |
| 申请人职称 |  | 申请人工作单位 |  |
| 申请人职位 |  | 申请人工作部门 |  |
| 申请人电子邮件 |  | 申请人电话 |  |
| 申请人通讯地址 |  | 申请目的 |  |
| 是否愿意与本机构共享使用本数据产生的研究成果的知识产权 | | | □是  □否 |
| 是否自愿遵守本数据保密协议 | | | □是  □否 |

National survey of Clinical Pharmacy Services in County Hospitals in China is a large-scale social research implemented by the State Development Research Center of Licensed Pharmacists in China Pharmaceutical University.

This research will collect and analyze the latest high-quality micro data aiming at present implementation situation of clinical pharmacy services in China’s county hospitals, and obtains information such as equipment, staffing, quality and status of servers, laying more scientific foundations for designing and improving Chinese national policies. Carried out in July 2015 to August 2015, the large-sample survey covers 317 county hospitals among all 31 provinces, municipalities and municipalities directly under the central government in mainland China. Based on related regulations and policies worldwide and international experiences of researches on clinical pharmacy services, the study applies complex multi-stage sampling to perform statistical analysis on data.

The survey is the first large-sample research covering all mainland counties on clinical pharmacy services of county hospitals in China, as well as the origin of a series of researches.

Please fill in the appendix, send it to our e-mail, and contact us if details about questionnaires and data are needed.

| Data acquisition application of National Survey of Clinical Pharmacy Services in County Hospitals in China | | | |
| --- | --- | --- | --- |
| Name of applicant |  | Institution of application |  |
| Professional title of applicant |  | Institution of applicant |  |
| Position of applicant |  | Department of applicant |  |
| E-mail of applicant |  | Telephone number |  |
| Address of applicant |  | Reason of application |  |
| Is the institution of application willing to share the intellectual property of the achievements in which the provided data is applied | | | □Yes  □No |
| Is the institution of application willing to obey the data security agreement | | | □Yes  □No |
